# MiR‐148b suppressed non‐small cell lung cancer progression via inhibiting ALCAM through the NF‐κB signaling pathway

**DOI:** 10.1111/1759-7714.13285

**Published:** 2019-12-27

**Authors:** Zhe Jiang, JingWen Zhang, FuHui Chen, Yajiao Sun

**Affiliations:** ^1^ Department of Pulmonary and Critical Care Medicine The Second Affiliated Hospital of Harbin Medical University Heilongjiang China

**Keywords:** ALCAM, miR‐148b, NF‐κB, NSCLC, progression

## Abstract

**Background:**

Non‐small cell lung cancer (NSCLC) is the leading cause of cancer mortality worldwide. MiRNAs are recognized as important molecules in cancer biology. The aim of the study was to identify a novel biomarker miR‐148b and its mechanism in the modulation of NSCLC progression.

**Methods:**

The expressional level of miR‐148b was analyzed by RT‐PCR. The effect of miR‐4317 on proliferation was evaluated through 3‐(4,5‐Dimethyl‐2‐thiazolyl)‐2,5‐diphenyl‐2Htetrazolium bromide (MTT) assay. The effect of miR‐148b on the metastasis of NSCLC was detected through transwell assays. The verification of the target of miR‐148b was assessed by TargetScan and dual‐luciferase reporter assay. The related proteins in this study were analyzed by western blot.

**Results:**

Our findings confirmed that miR‐148b was decreased in NSCLC and NSCLC patients with lower expression exhibited poorer overall survival (OS). Increasing miR‐148b significantly repressed proliferation, invasion and migration. More importantly, activated leukocyte cell adhesion molecule (ALCAM) was determined as the direct target of miR‐148b, and reintroduction of ALCAM attenuated miR‐148b effect on the progress of NSCLC. In addition, NF‐κB signaling pathway was modulated by miR‐148b/ALCAM axis.

**Conclusions:**

Our results indicated that miR‐148b is able to suppress NSCLC growth and metastasis via targeting ALCAM through the NF‐κB pathway. These findings provided new evidence that miR‐148b serves as a potential biomarker and novel target for NSCLC treatment.

## Introduction

Lung cancer, one of the most common malignant tumors worldwide, has become the first cause of malignant tumor‐associated death in China.^1^ Non‐small cell lung cancer (NSCLC) accounts for about 80% of all lung cancer. Since approximately 75% of patients are diagnosed at late stage, the five‐year survival rate is very low.^2^, ^3^ The poor outcomes and frequent relapses associated with lung cancer urgently demand the development of new screening methods and early biomarkers for accurate and noninvasive detection of lung cancer metastasis and recurrence.^4^, ^5^ Thus, new screening methods and early biomarkers to accurately detect, prevent and treat NSCLC is urgently required.

MiRNAs have been reported to take part in several biological processes such as proliferation, differentiation, and apoptosis.^6^, ^7^ Recent studies have suggested that miRNAs play critical roles in multiple cancers including lung cancer, melanoma, and prostate cancer.^8^, ^9^, ^10^ Increasing findings show that several miRNAs exhibited important roles in the tumorigenesis of NSCLC as oncogenes or tumor suppressors. For instance, upregulation of miR‐221, −21, −421^11^, ^12^, ^13^ were found in NSCLC and participated in the proliferation, metastasis and apoptosis as oncogenes. Moreover, downregulation of miR‐612, −621, −34b have been proven to be associated with the development of NSCLC.^14^, ^15^, ^16^ Wang *et al*. reported that miR‐148b was underexpressed in NSCLC and served as a potential prognostic biomarker.^17^ Lu *et al*. demonstrated that miR‐148b modulated tumor growth in NSCLC.^18^ However, the biological function of miR‐148b and its mechanism in the modulation of NSCLC invasion and migration has not been fully clarified.

MiRNAs have been reported to regulate gene expression through repression of mRNA stability or translation and their role in cell growth and differentiation. Activated leukocyte cell adhesion molecule (ALCAM) is a member of the immunoglobulin super‐family and acts as an intercellular adhesion molecule. ALCAM is receiving more and more attention because of its important relationship with tumor progression and metastasis.^19^ Ferragut *et al*. reported the significant role of ALCAM in controlling triple negative breast cancer^.^ 20 He *et al*. showed that inhibition expression of ALCAM repressed proliferation and invasion of pituitary adenomas cells.^21^ Moreover, Ishiguro and his colleagues revealed that knockdown of ALCAM suppressed NSCLC cell migration and invasion and that it was associated with the prognosis of NSCLC patients.^22^ Based on these studies, we further investigated the effect of ALCAM on miR‐148b in regulating NSCLC invasion and migration.

Nuclear factor κB (NF‐κB) is a nuclear transcription factor which regulates expression of a large number of genes critical for the regulation of apoptosis, tumorigenesis, inflammation, and various autoimmune diseases.^23^, ^24^, ^25^ The NF‐κB signaling pathway is modulated by several miRNAs in certain types of cancers as previously reported.^26^, ^27^ Thus, we examined whether the NF‐κB signaling pathway was regulated by miR‐148b in NSCLC.

The purpose of our study was to explore miR‐148b effect on NSCLC invasion and migration and to investigate the potential mechanism of miR‐148b in the progression of NSCLC.

## Methods

### NSCLC tissue specimens

A total of 70 paired NSCLC tissue specimens and nontumor tissues were obtained from the Second Affiliated Hospital of Harbin Medical University from March 2014 to July 2017. Table 1 summarizes the histologic characteristics of NSCLC patients. The patients included in this study did not receive any treatment prior to surgery and provided their written informed consents. The experimental methods were conducted in strict accordance with the guidelines of the experimental procedure approved by The Second Affiliated Hospital of Harbin Medical University. The tissues were stored at −80°C until further use.

**Table 1:** Associations between miR‐148b expression and clinicopathological characteristics

		miR‐148b		
Characteristics	*n* = 70	High	Low	*P*‐value
Age (years)				0.281
≥60	28	15	13	
<60	42	17	25	
Gender				0.806
Male	33	22	21	
Female	37	13	14	
Smoker				0.331
Yes	34	14	20	
No	36	19	17	
Histology				0.885
LSC	25	11	14	
LAC	45	19	26	
Tumor size				0.002*
≥5 cm	29	7	22	
<5 cm	41	25	16	
TNM stage				0.017*
I–II	41	15	26	
III–IV	29	19	10	
Lymph node metastasis				0.026*
Absence	22	15	7	
Presence	48	19	29	

* *P* < 0.05 was considered significant.

Statistical analyses were performed by the χ^2^ test.

### Cell culture

NSCLC cells (A549 and H1299) and human immortalized normal epithelial cell (BEAS‐2B) used in this study were grown in RPMI 1640 medium containing 10% FBS and L‐glutamine at 37°C with 5% CO_2_ atmosphere. All cell lines were purchased from the ATCC (USA).

### Cell transfection

MiR‐148b mimic, inhibitor, ALCAM siRAN and their corresponding negative controls were provided by RiboBio (Guangzhou, China). The transfection was performed with the assistance of Lipofectamine 2000 (Invitrogen, Waltham, MA, USA). After 48 hours transfection, the cells were collected for further studies.

### RT‐PCR

TRIzol reagent (Invitrogen) was used to extract total RNA from patient samples and cultured cells. Bulge‐Loop miRNA‐specific RT primers or random primers with MMLV reverse transcriptase were used for reverse transcription. The Step One Plus real‐time system was applied for quantitative RT‐PCR reactions. U6 or GAPDH were used as internal controls. The 2^−ΔΔ 2^ method was used for relative quantification. The primers used are listed in Table 2.

**Table 2:** Primer sequences for RT‐qPCR

Primer	Sequence
miR‐148b forward	5′‐ ACACTCCAGCTGGGTCAGTGCATC‐3′
Reverse	5′‐ CTCAACTGGTGTCGTGGA‐3′
U6 forward	5′‐ CTCGCTTCGGCAGCACA −3′
Reverse	5′‐AACGCTTCACGAATTTGCGT‐3′
ALCAM forward	5′‐TCAATGGACAATTACTGGCAG‐3′
Reverse	5′‐AGTTGGTTTTCTGCTGTGC‐3′
GAPDH forward	5′‐CTCTGATTTGGTCGTATTGGG‐3′
Reverse	5′‐TGGAAGATGGTGATGGGATT‐3′

ALCAM, activated leukocyte antigen molecule; GAPDH, glyceraldehyde‐3‐phosphate dehydrogenase; U6, small nuclear RNA, snRNA.

### Western blot analysis

The whole‐cell lysates were prepared using RIPA buffer containing protease inhibitors. After protein concentration by BCA kit, equal amounts of proteins were added onto SDS‐PAGE, followed by PVDF membranes. Subsequently, the membranes were blocked with 5% bovine serum albumin for one hour, primary antibodies at 4°C overnight, and then secondary antibodies for one hour. Image Reader LAS‐4000 and Multi Gauge V3.2 software were applied for visualizing and analyzing the proteins.

### MTT assay

MTT assay was carried out for testing cell proliferation. First, 4 × 10^3^ cells/well were seeded into 96‐well plates and 200 μL of medium containing 10% fetal calf serum added to each well. The cells were then cultured for one, two, three, and four days and MTT reagent (20 μL, Sigma‐Aldrich, St. Louis, MO, USA) was added for incubation for another four hours. Next, the medium was removed and 150 μL DMSO was added to dissolve crystals. Finally, a microplate reader was used for measuring the optical density at a wavelength of 490 nm.

### Transwell invasion/migration assay

Transwell chambers (8 μM pore size) were utilized to perform the cell migration assay and 5 × 10^4^ cells were added into the upper chamber and the complete medium into the lower chamber. After incubation for 24 hours, the cells in the upper chamber that had not migrated were removed using cotton swabs. The migrated cells were fixed with methanol and then stained with crystal violet. Five images of different fields of view were captured for each film, and the number of migrated cells was counted. A similar insert coated with Matrigel was used to assess cell invasiveness in the invasion assay.

### Luciferase reporter assay

The wild‐ or mutated type of ALCAM 3'UTR, known as ALCAM WT or ALCAM MT was generated and inserted into the psiCHECK‐2 luciferase reporter plasmid (Promega, Woods Hollow Road, USA). A549 and NCI‐H1299 cells were cultured for 24 hours and then treated with ALCAM WT or MT 3'UTR reporter and miR‐148b mimic for transfection using Lipofectamine 2000 reagent. Dual Luciferase Reporter Assay System was employed to check the luciferase activity. All luciferase activity data are presented as means ± SD from at least three independent experiments.

### Statistical analysis

Each independent experiment was repeated at least three times and the results shown as mean ± SD. Statistical analysis was performed by SPSS v.19.0 software and graph presentations by GraphPad Prism 6 software. *P*‐value significance was compared using Student's *t*‐test or Tukey's post hoc test. A statistically significant difference was considered as *P* < 0.05.

## Results

**Figure 1:**
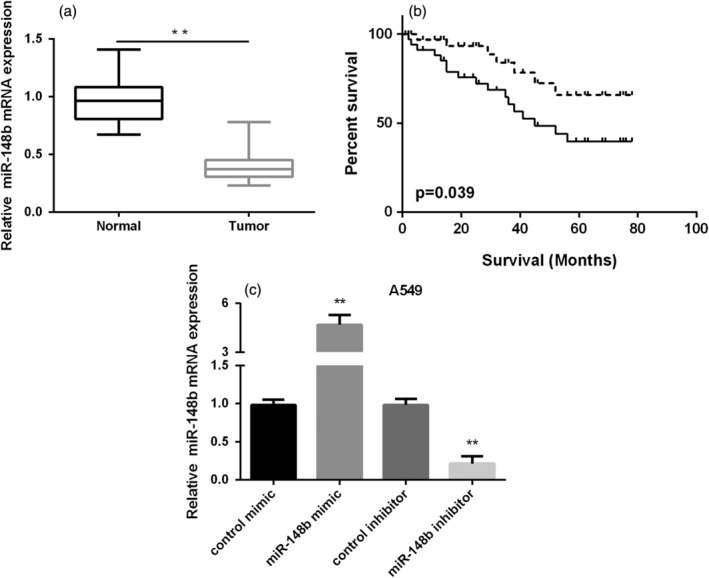
MiR‐148b expression in NSCLC tissues and its clinical significance. (**a**) Relative expressional level of miR‐148b in NSCLC tissues. (**b**) Kaplan‐Meier overall survival curve of low or high expression of miR‐148b in patients with NSCLC (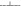) High miR‐148b, and (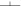) Low miR‐148b. (**c**) miR‐148b mRNA expression in NSCLC cells.

To identify whether miR‐148b was associated with the overall survival of NSCLC patients, we examined the miR‐148b expression in NSCLC tissue specimens of 70 patients. The findings indicated that miR‐148b was underexpressed in NSCLC tissues (Fig 1a). More importantly, it was found that miR‐148b was closely associated with the prognosis of NSCLC patients, based on Kaplan‐Meier survival analysis (Fig 1b). The patients with high expression of miR‐148b were predicted to have better overall survival in comparison with the patients with low expression of miR‐148b. Furthermore, as summarized in Table 1, miR‐148b was significantly associated with clinical stage, tumor size and lymph node metastasis. However, there was no difference in age, gender and histology. These findings indicated that the lower expression of miR‐148b was associated with the clinicopathologic features of NSCLC patients.

### MiR‐148b repressed NSCLC proliferation, invasion and migration

**Figure 2:**
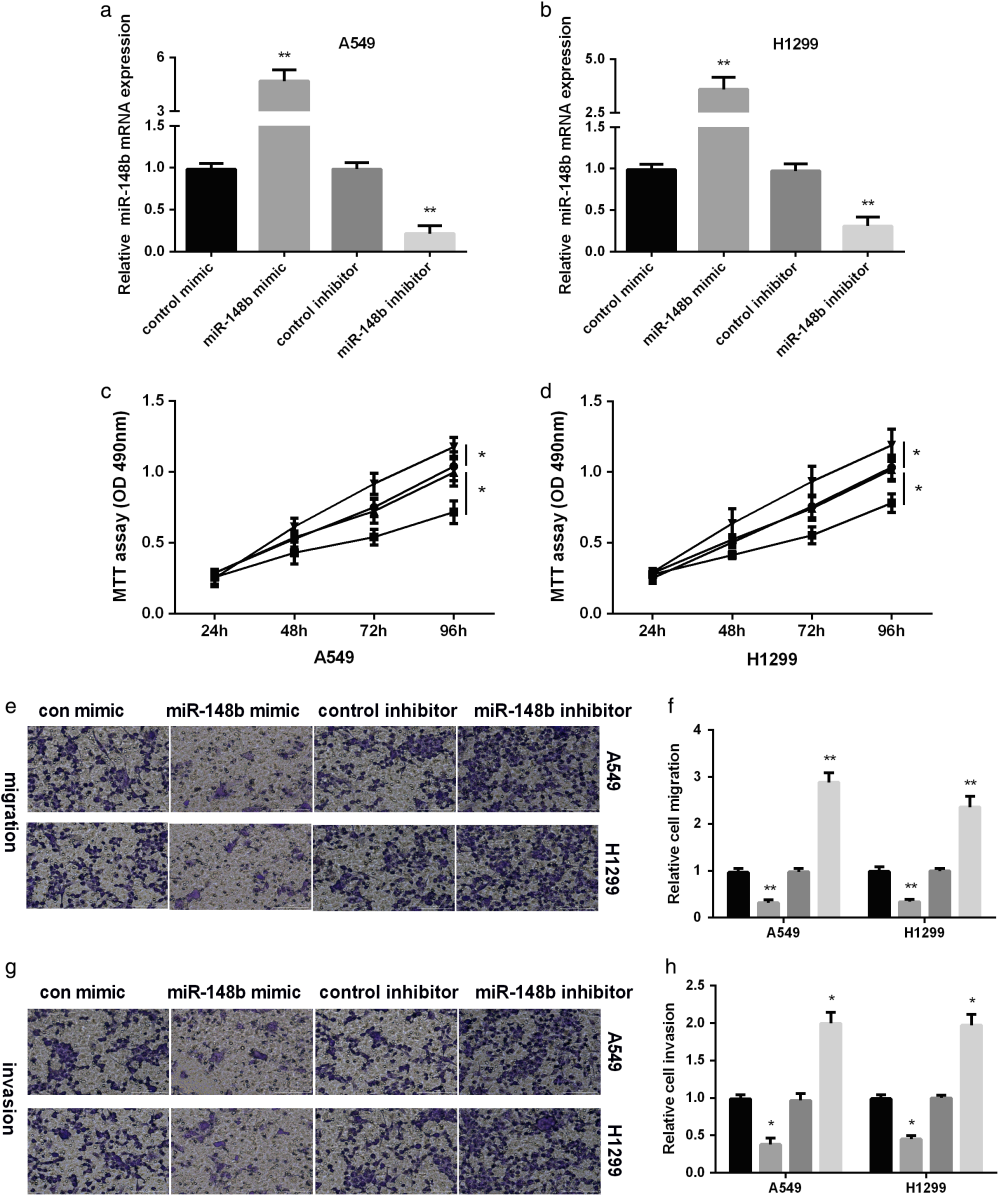
MiR‐148b inhibited NSCLC migration and invasion. (**a**) Measurement of miR‐148b level after treated with miR‐148b mimic or inhibitor in NSCLC cells. (**b**) A549 and H1299 cells viability measured after treated with miR‐2861 mimic or miR‐2861 inhibitor by MTT (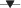) miR‐148b inhibitor, (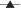) control inhibitor, (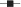) miR‐148b mimic, and (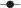) control mimic. (**c**) Representative images and quantitation of cell migration and (**d**) cell invasion after re‐expression or knockdown of miR‐148b in NSCLC cells. (**c**, **d**) (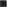) control mimic, (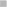) miR‐148b mimic, (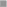) control inhibitor and (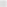) miR‐148b inhibitor.

To assess the role of miR‐148b in NSCLC, we initially detected miR‐148b expression in NSCLC cells (A549 and H1299 cells). As evidenced in Fig 1c, miR‐148b was decreased remarkably in NSCLC cells compared to normal epithelial cells (BEAS‐2B). Next, A549 and H1299 cells were transfected with miR‐148b mimic or inhibitor to overexpression or knockdown of miR‐148b expression. The expressional level of miR‐148b was significantly increased in both NSCLC cells after treated with miR‐148b mimic, while decreased after treated with miR‐148b inhibitor, demonstrating that the efficiency of transfection was very successful (Fig 2a). The results of MTT assays showed that overexpression of miR‐148b inhibited A549 and H1299 cell viability and knockdown of miR‐148b promoted A549 and H1299 cell viability as shown in Fig 2b. Transwell migration assay results indicated that high expression of miR‐148b suppressed cell migration, whereas low expression of miR‐148b enhanced cell migration in both NSCLC cells (Fig 2c). Moreover, we also observed that increasing miR‐148b expression could inhibit the ability of cell invasiveness, while miR‐148b inhibitor showed the opposite results in two NSCLC cells (Fig 2d). These findings indicated that miR‐148b acted as a suppressor on the capacity of NSCLC cell proliferation and metastasis.

### ALCAM a target of miR‐148b in NSCLC cells

**Figure 3:**
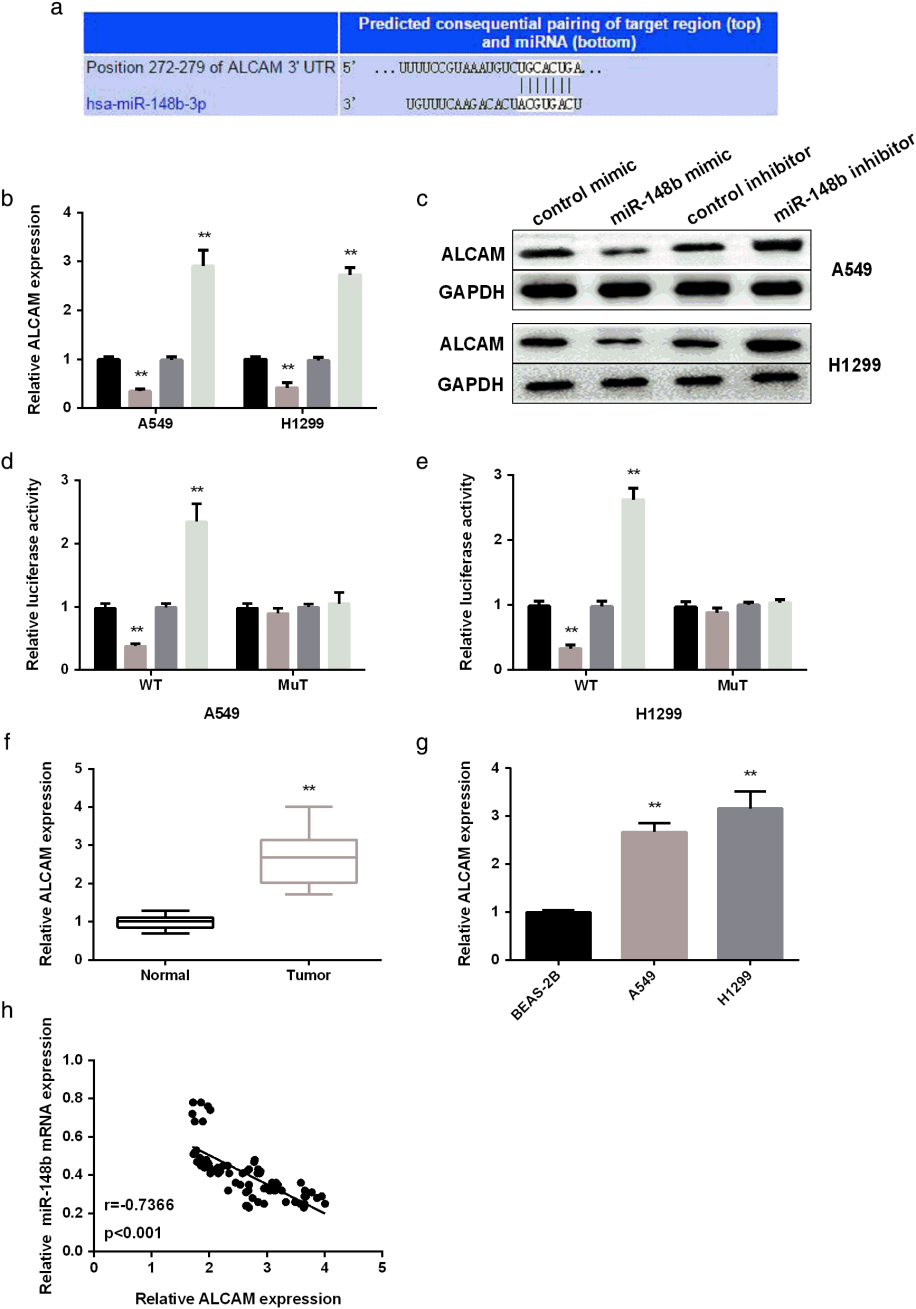
ALCAM was the target of miR‐148b. (**a**) Prediction binding sites of ALCAM containing with miR‐148b. (**b**,**c**) ALCAM mRNA and protein level detected in NSCLC cells after re‐expression or knockdown of miR‐148b. (**d**,**e**) Relative luciferase activity in NSCLC cells after transfection with miR‐148b mimic or inhibitor. (**f**) RT‐PCR analysis of ALCAM level in NSCLC tissues. (**g**) RT‐PCR analysis of ALCAM level in NSCLC cell lines. (**h**) Repression analysis of correlation of miR‐148b with ALCAM. (**b**, **c**, **e**) (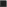) control mimic, (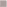) miR‐148b mimic, (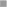) control inhibitor and (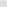) miR‐148b inhibitor.

To investigate the mechanism of miR‐148b in the regulation of NSCLC development, we searched for the possible target of miR‐148b using TargetScan. As seen in Fig 3a, ALCAM was determined as the candidate target of miR‐148b. To verify whether ALCAM was the direct target of miR‐148b, miR‐148b mimic or inhibitor was transfected into two NSCLC cells and the results indicated that miR‐148b restoration significantly reduced ALCAM mRNA and protein levels, while increased with miR‐148b inhibitor (Fig 3b,c). Dual‐luciferase reporter assay was then applied to reveal the manner by which miR‐148b regulated ALCAM. As show3d,e, miR‐148b mimic significantly repressed, while miR‐148b inhibitor facilitated the luciferase activities of ALCAM 3'UTR WT. However, the luciferase activities of ALCAM 3'UTR MUT did not show any changes after re‐expression or knockdown of miR‐148b. In addition, RT‐PCR results showed high ALCAM expression in NSCLC tissues and cells compared to normal controls (Fig 3f,g). The relationship between miR‐148b and ALCAM expression was negative (Fig 3h). These data suggest that miR‐148b is able to modulate ALCAM expression by directly targeting its 3'UTR.

### ALCAM overturned miR‐148b effect on NSCLC metastasis

**Figure 4:**
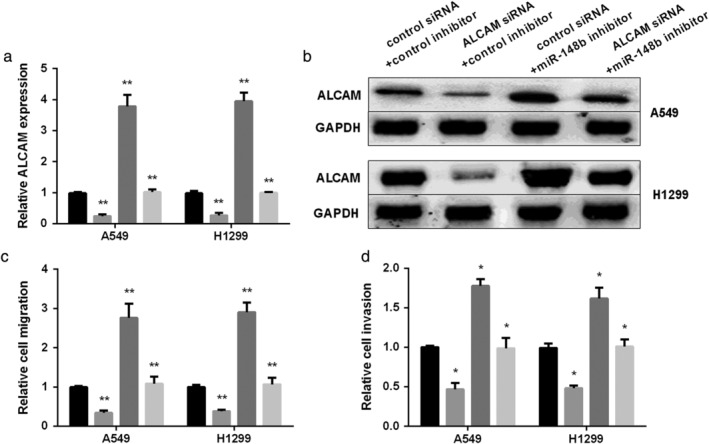
Rescue effect of ALCAM on miR‐148b in inhibiting NSCLC migration and invasion. (**a** and **b**) ALCAM mRNA and protein level tested in NSCLC cells cotransfected with miR‐148b inhibitor and ALCAM siRNA. (**c**) Representative images and quantitation of cell migration and (**d**) cell invasion after re‐expression of miR‐148b or both miR‐148b and ALCAM in NSCLC cells. (**a**, **c**, **d**) (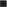) control siRNA+control inhibitor, (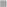) ALCAM siRNA+control inhibitor, (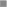) control siRNA+miR‐148b inhibitor, and (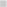) control siRNA+miR‐148b inhibitor.

To access the effect of ALCAM on miR‐148b in regulating NSCLC invasion and migration, a rescue experiment was performed. Western blot and RT‐PCR assays were initially applied to detect ALCAM expression affected by miR‐148b. The protein and mRNA level of ALCAM was reduced by ALCAM siRNA and recovered by cotransfection with miR‐148b inhibitor and ALCAM siRNA in two NSCLC cells (Fig 4a,b). Furthermore, transwell analysis results displayed that the migration of NSCLC cells elevated by miR‐148b inhibitor was reversed by transfection of miR‐148b inhibitor and ALCAM siRNA (Fig 4c). Also, ALCAM siRNA rescued the facilitating effect of miR‐148b inhibitor on NSCLC invasion (Fig 4d). These findings revealed that ALCAM reversed miR‐148b effect on NSCLC metastasis.

### MiR‐148b/ALCAM axis regulated EMT and NF‐κB signaling pathway in NSCLC cells

**Figure 5:**
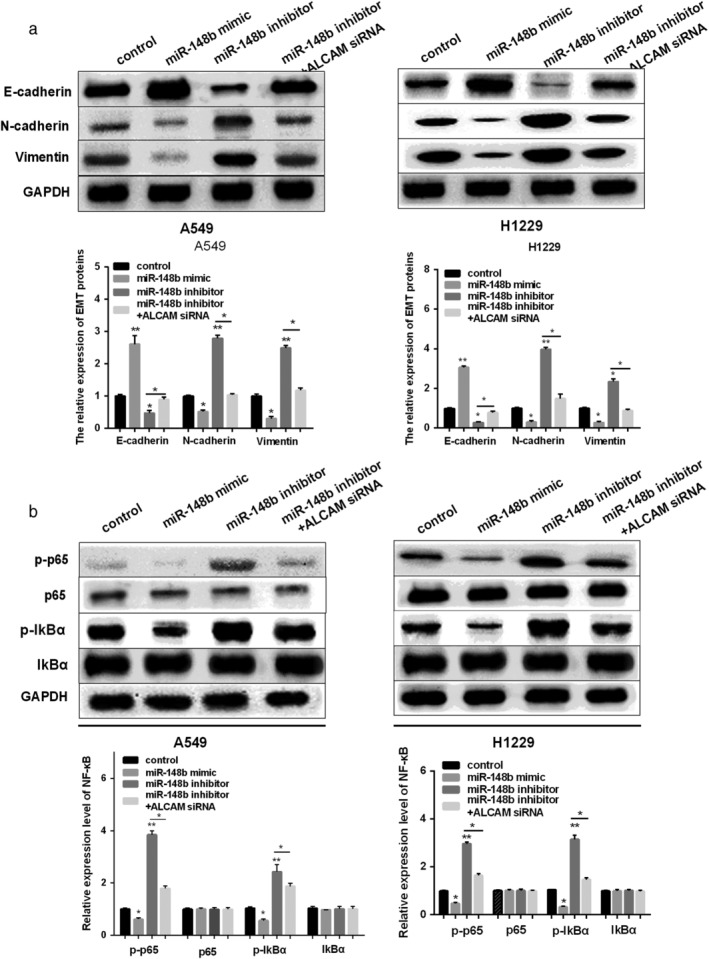
MiR‐148b/ALCAM axis regulated EMT and NF‐κB pathway in NSCLC cells. (**a**) E‐cadherin, N‐cadherin and Vimentin expression detected in NSCLC cells after re‐expression of miR‐148b or both miR‐148b and ALCAM. (**b**) p65/p‐p65 and p‐IkBα/IkBα expression detected in NSCLC cells after re‐expression of miR‐148b or both miR‐148b and ALCAM by RT‐PCR analysis and western blot ALCAM. (**a**, **b**) (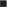) control, (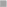) miR‐148b mimic, (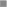) miR‐148b inhibitor and (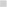) miR‐148b inhibitor+ALCAM siRNA.

To further explore whether EMT and NF‐κB pathway were involved in the development of NSCLC regulated by miR‐148b/ALCAM axis, the downstream genes of NF‐κB pathway and EMT‐related markers were detected by western blot. As shown in Fig 5a, the expression of E‐cadherin was increased by miR‐148b mimic, while miR‐148 inhibitor decreased the expression of E‐cadherin. However, miR‐148b mimic decreased, while miR‐148b inhibitor increased N‐cadherin, Vimentin expression. Silencing ALCAM could overturn miR‐148b inhibitor effect on EMT‐related markers. Furthermore, miR‐148b mimic inhibited the activation of NF‐κB pathway, while miR‐148b inhibitor promoted the activation of NF‐κB pathway. ALCAM siRNA also overturned the promotion effect of miR‐148b inhibitor on the NF‐κB pathway (Fig 5b). Correctively, miR‐148b/ALCAM axis regulated EMT and the NF‐κB signaling pathway in NSCLC.

## Discussion

In this study, we found a decreased expression of miR‐148b in NSCLC and its low expression was closely associated with the prognosis of NSCLC patients. Moreover, restoration of miR‐148b repressed NSCLC cell proliferation, invasion and migration, while inhibiting expression of miR‐148b enhanced NSCLC cell proliferation, invasion and migration. More strikingly, ALCAM was determined as the direct target of miR‐148b and it rescued the inhibitory effect of miR‐148b on NSCLC metastasis. In addition, we demonstrated that miR‐148b blocked the activation of the NF‐κB signaling pathway by inhibiting ALCAM, resulting in suppression of invasion and migration.

Recently, it has been identified that miRNAs are linked to the proliferation and metastasis of NSCLC. Kang *et al*. reported that miR‐612 suppressed the malignant development of NSCLC by targeting BRD4 through the PI3K/Akt pathway.^14^ Feng and colleagues reported that miR‐34b exhibited a repressive effect on NSCLC cell proliferation and promotion effect on cell apoptosis^.^ 16 MiRNAs have drawn great attention to the diagnosis, prognosis and metastasis of tumors. Here, in our study, miR‐148b expression was found to be downregulated in NSCLC and was closely associated with clinical significance. More importantly, the low expression of miR‐148b might predict the poor survival of NSCLC patients. The results were in line with the reports that miR‐148b functions as a potential prognostic biomarker in NSCLC.^17^

Metastasis‐related deaths in tumors account for approximately 90% of total mortality.^28^ Increasing evidence has shown that miRNAs are involved in the proliferation and metastasis of tumors, including lung cancer^.^ 29, 30 Zhou and Li demonstrated that miR‐605 restoration hindered the metastasis of NSCLC.^31^ Upregulation of miR‐340 repressed tumor growth and metastasis of NSCLC in the study by Lu and Zhang.^32^ Similarly, miR‐150 exhibited an inhibitory effect on the metastasis of NSCLC.^33^ Here, we revealed that miR‐148b upregulation hindered the proliferation, invasion and migration of NSCLC. In contrast, miR‐148b downregulation displayed the opposite effect on NSCLC proliferation and metastasis. These results indicate that miR‐148b plays an important role in the development of NSCLC.

The mechanism of miRNAs altered gene expression by targeting their mRNA. Our bioinformatics analysis indicated that miR‐148b and ALCAM had binding sites, and miR‐148b could negatively regulate the expression of ALCAM. ALCAM was reported to take part in tumor progression and metastasis as the target of miRNAs. For instance, it served as the target of miR‐483 in the regulation of hepatocellular carcinoma.^34^ He *et al*. revealed that miR‐152/ALCAM axis modulated pituitary adenomas cells proliferation and invasion.^21^ More importantly, in lung adenocarcinama, ALCAM acted as the target of miR‐483 in regulating invasion and metastasis.^35^ In this study, we first proved ALCAM was the direct target of miR‐148b in inhibiting NSCLC invasion and migration. More interestingly, ALCAM could rescue the suppressive effect of miR‐148b on the metastasis of NSCLC.

To better understand the precise mechanism of miR‐148b in the development of NSCLC, we explored whether NF‐κB signaling pathway participated in the metastasis of NSCLC modulated by miR‐148b/ALCAM axis. Here, we showed that re‐expression of miR‐148b hindered the activation of NF‐κB pathway, while knockdown of miR‐148b exhibited the opposite effect. Moreover, ALCAM reversed the effect of miR‐148b on NF‐κB pathway in NSCLC cells.

In conclusion, these findings demonstrated that miR‐148b suppressed NSCLC progression via inhibiting ALCAM through NF‐κB signaling pathway.

## Funding

This study was supported by the National Major Science and Technology Project for the Control and Prevention of Major Infectious Diseases of China (2017ZX10103004) and Natural Science Foundation of Heilongjiang Province of China (LH2019H071).

## Disclosure

No authors report any conflict of interest.
